# Incorporating significant amino acid pairs to identify O-linked glycosylation sites on transmembrane proteins and non-transmembrane proteins

**DOI:** 10.1186/1471-2105-11-536

**Published:** 2010-10-29

**Authors:** Shu-An Chen, Tzong-Yi Lee, Yu-Yen Ou

**Affiliations:** 1 Department of Computer Science and Engineering, Yuan Ze University, Chungli 320, Taiwan

## Abstract

**Background:**

While occurring enzymatically in biological systems, O-linked glycosylation affects protein folding, localization and trafficking, protein solubility, antigenicity, biological activity, as well as cell-cell interactions on membrane proteins. Catalytic enzymes involve glycotransferases, sugar-transferring enzymes and glycosidases which trim specific monosaccharides from precursors to form intermediate structures. Due to the difficulty of experimental identification, several works have used computational methods to identify glycosylation sites.

**Results:**

By investigating glycosylated sites that contain various motifs between Transmembrane (TM) and non-Transmembrane (non-TM) proteins, this work presents a novel method, GlycoRBF, that implements radial basis function (RBF) networks with significant amino acid pairs (SAAPs) for identifying O-linked glycosylated serine and threonine on TM proteins and non-TM proteins. Additionally, a membrane topology is considered for reducing the false positives on glycosylated TM proteins. Based on an evaluation using five-fold cross-validation, the consideration of a membrane topology can reduce 31.4% of the false positives when identifying O-linked glycosylation sites on TM proteins. Via an independent test, GlycoRBF outperforms previous O-linked glycosylation site prediction schemes.

**Conclusion:**

A case study of Cyclic AMP-dependent transcription factor ATF-6 alpha was presented to demonstrate the effectiveness of GlycoRBF. Web-based GlycoRBF, which can be accessed at http://GlycoRBF.bioinfo.tw, can identify O-linked glycosylated serine and threonine effectively and efficiently. Moreover, the structural topology of Transmembrane (TM) proteins with glycosylation sites is provided to users. The stand-alone version of GlycoRBF is also available for high throughput data analysis.

## Background

Protein glycosylation adds an oligosaccharide (chain of sugars) to a polypeptide (chain of amino acids) in order to produce a glycoprotein. Many proteins in eukaryotic cells are glycoproteins since they contain oligosaccharide chains covalently linked to certain amino acids. Among the predominant sugars found in glycoproteins include xylose, fucose, galactose, glucose, mannose, N-acetylglucosamine, N-acetylgalactosamine, and N-acetylneuraminic acid [1]. Specific carbohydrate epitopes can serve as ligands for receptors that mediate recognition events [2]. Catalytic enzymes involve glycotransferases, sugar-transferring enzymes and glycosidases which trim specific monosaccharides from precursors to form intermediate structures [3]. As is well known, glycosylation affect protein folding, localization and trafficking, protein solubility, antigenicity, and biological activity, as well as cell-cell interactions on membrane proteins [4]. Protein glycosylation can be divided into four main categories mainly depending on the linkage between the amino acid and the sugar, including N-linked glycosylation, O-linked glycosylation, C-mannosylation and glycophosphatidlyinositol (GPI) anchor attachments [5].

Owing to the difficulty of experimental identification, several works have computationally identified glycosylation sites, which could be a feasible means of conducting preliminary analyses and significantly reducing the number of potential targets that require further *in vivo *or *in vitro *confirmation. Blom *et al*. [6] are the first group to propose a method for computationally identifying glycosylation sites. Gupta *et al*. [7] have proposed a web-based tool, named NetNGlyc, for identifying N-glycosylation sites in human proteins. Li *et al*. [8] applied support vector machine (SVM) for predicting O-glycosylation sites in mammalian proteins. Caragea *et al*. [9] have used the ensembles of SVM classifiers to predict glycosylation sites. Additionally, NetOglyc3.1 [2], CKSAAP [10], and GPP [11] are well-maintained O-linked glycosylation prediction web servers. Julenius *et al*. [2] combined sequence, surface accessibility, secondary structure, and distance constraints for building neural network glycosylation prediction model. Chen *et al*. [10] analyzed the *k*-spaced amino acid pairs of glycol-proteins and developed support vector machine based method to predict O-link glycosylation sites. Hamby and Hirst [11] adopted the random forests method, integrating frequencies of amino acids surrounding modified residue and significant pairwise patterns for predicting glycosylation site.

By investigating glycosylated sites that contain various motifs between transmembrane (TM) proteins and non-transmembrane (non-TM) proteins, this work presents a novel method, GlycoRBF, that implements radial basis function (RBF) networks for identifying O-linked glycosylated sites on TM proteins and non-TM proteins. Based on statistical measurement, the significant amino acid pairs (SAAPs) that surround O-linked glycosylation sites are adopted to improve the prediction performance. By combining the identified SAAPs with the sequence of amino acids, the predictive specificity for glycosylated TM proteins and non-TM proteins is greatly improved; this is based on an evaluation using five-fold cross validation. Additionally, a membrane topology is considered for reducing the false positives on glycosylated TM proteins. Via an independent test, GlycoRBF outperforms previously published glycosylation site prediction schemes. To investigate the characteristics of O-linked glycosylation sites in a comprehensive manner, 531 physicochemical properties, that were extracted from version 9.1 of AAindex [12], were evaluated for its ability to distinguish the glycosylation sites from the non-glycosylation sites. To demonstrate the performance of GlycoRBF, a case study of Cyclic AMP-dependent transcription factor ATF-6 alpha was presented. Web-based GlycoRBF, which can be accessed at http://GlycoRBF.bioinfo.tw, can identify O-linked glycosylated serine and threonine effectively and efficiently. Moreover, the membrane topology of TM proteins with glycosylation sites is provided to users. The stand-alone version of GlycoRBF is also available for high throughput data analysis.

## Methods

**Figure 1:**
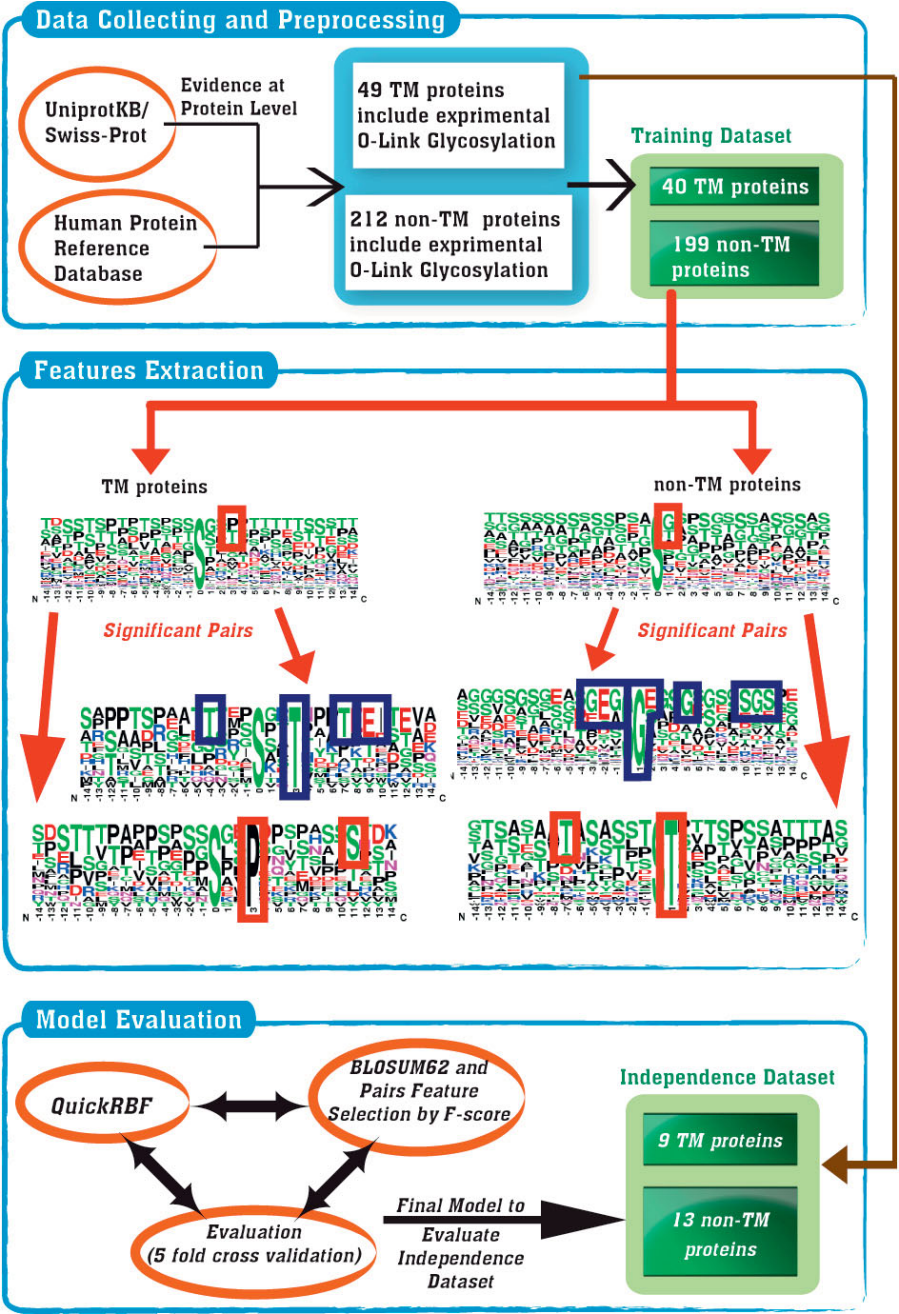
**The analyzing flowchart of GlycoRBF**.

According to Figure 1, the analyzing flowchart consists of collecting and preprocessing data, extracting features, and learning and evaluating models. This work focuses on identifying O-linked glycosylation sites on Transmembrane (TM) and non-Transmembrane (non-TM) proteins. Following the model learning and evaluation, the selected models which contain the highest predictive accuracy are tested on an independent data set. All of the detailed processes are described as follows.

### Data collection and preprocessing

Release 15.0 of UniProt [13], which is the universal knowledge base of proteins, consists of 839 experimentally verified O-linked glycosylation sites in 239 glycoproteins. As shown in Table 1, 202 and 637 O-linked glycosylation sites are located on 40 TM proteins and 199 non-TM proteins, respectively. The O-linked glycosylation sites occurred mainly on residues of serine and threonine. In this work, all experimental O-linked glycosylated serine and threonine collected from UniProt are regarded as positive set of the original data. The amino acids of serine and threonine that have not been annotated as glycosylation sites on the experimental O-linked glycoproteins are regarded as negative set of the original data. Consequently, 2450 and 14234 non-glycosylation sites are located on TM proteins and non-TM proteins, respectively. The significant amino acid pairs (SAAPs) around the O-linked glycosylation sites are identified based on the original data. These SAAPs are then adopted to construct the radial basis function (RBF) networks for differentiating O-linked glycosylation sites from non-glycosylation sites on transmembrane and non-transmembrane proteins. To prevent overestimation of the predictive performance, homologous sequences are removed from the dataset by using a window size of 2*n*+1 for O-linked glycosylation sites. With reference to the reduction of the homology in the original dataset of MASA [14], two glycosylated protein sequences with more than 30% identity were defined as homologous sequences. Then, two homologous sequences were specified to re-align the fragment sequences using a window length of 2*n*+1, centered on the glycosylated sites using BL2SEQ [15]. For two fragment sequences with 100% identity, when the glycosylated sites in the two proteins are in the same positions, only one site was kept while the other was discarded. The non-homologous negative data were generated using the same approach as positive one.

**Table 1:** The statistics of experimentally verified O-linked glycosylation sites on transmembrane (TM) proteins and non-transmembrane (non-TM) proteins.

Database	Original data (UniProt release 15.0)		Independent test data (HPRD release 8.0)	
	
	TM proteins	non-TM proteins	TM proteins	non-TM proteins
Number of O-linked glycosylated proteins	40	199	9	13

Number of O-linked glycosylated serine	87	281	7	10

Number of O-linked glycosylated threonine	115	356	7	34

Number of non-glycosylated serine and threonine	2,450	14,234	1,238	662

The experimental O-linked glycosylated proteins extracted from UniProt release 15.0 are regarded as the original data. To evaluate the real performance of the constructed models, the experimental O-linked glycosylated proteins collected from HPRD release 8.0 are used for independent testing.

As for classification, the prediction performance of the trained models may be overestimated owing to the over-fitting of a training set. The experimental O-linked glycosylation sites of HPRD release 8.0 [16], which were not included in UniProt [13], are regarded as the independent test set and are used to estimate the actual prediction performance. According to Table 1, 4 and 44 O-linked glycosylation sites are located on 9 TM proteins and 13 non-TM proteins, respectively. Similar to the extraction of a negative set of training data, a total of 1,238 and 662 non-glycosylated serines/threonines on TM proteins and non-TM proteins, respectively, are regarded as a negative set of independent test data. After the evaluation using *k*-fold cross-validation, the independent test set is evaluated by using the trained model with the highest accuracy. Independent test sets are utilized not only to demonstrate the effectiveness of the proposed method, but also to evaluate the performances of other previously proposed O-linked glycosylation prediction schemes.

### Feature extraction

Fragments of amino acids are extracted from positive and negative training sets using a window of length 2*n*+1 that is centered on O-linked glycosylation sites. Different values of *n *are used to determine the optimal window length. BLOSUM62 matrix is adopted to represent the protein primary sequence information as the basic feature set for learning radial basis function networks. A matrix of (2*n+*1) × *m *elements is used to represent each residue of a training dataset, where 2*n+*1 stands for the window size and *m *consists of 21 elements including 20 types of amino acids and one for terminal signal. Each row of the normalized BLOSUM62 matrix is utilized to encode each type of 20 amino acids.

In order to make further investigation of substrate site specificity, the significant amino acid pairs between transmembrane (TM) and non-transmembrane (non-TM) glycoproteins are identified based on statistical measurement of F-score [17]. Each position surrounding glycosylation site is calculated a value of F-score. The F-score of the *i*th feature is defined as:

(1) $$\text{F-score}(\text{i})=\frac{{\left({\bar{x}}_{i}^{\left(+\right)}-{\bar{x}}_{i}\right)}^{2}+{\left({\bar{x}}_{i}^{\left(-\right)}-{\bar{x}}_{i}\right)}^{2}}{\frac{1}{n^{+}-1}\sum_{k=1}^{n^{+}}{\left(x_{k,i}^{\left(+\right)}-{\bar{x}}_{i}^{\left(+\right)}\right)}^{2}+\frac{1}{n^{-}-1}\sum_{k=1}^{n^{-}}{\left(x_{k,i}^{\left(-\right)}-{\bar{x}}_{i}^{\left(-\right)}\right)}^{2}}$$

where ${\bar{x}}_{i}$, ${\bar{x}}_{i}^{\left(+\right)}$, and ${\bar{x}}_{i}^{\left(-\right)}$, denote the average value of the *i*th feature in whole, positive, and negative data sets, respectively; *n*^+ ^denotes the number of positive data set and *n*^- ^denotes the number of negative data set; $x_{k,i}^{\left(+\right)}$

denotes the *i*th feature of the *k*th positive instance, and $x_{k,i}^{\left(-\right)}$ denotes the *i*th feature of the *k*th negative instance [17].

After the calculation of the F-score value in each position around the glycosylated site, the significant position with highest F-score value is regarded as the starting point for extracting the significant amino acid pairs. As shown in Figure 1, the most frequent amino acids in significant position are determined and used to detect another frequent amino acid which is located on the other position. With the determination of one frequent amino acid on a specific position, another amino acid on a different position can be defined to generate the amino acid pair. After the detection of all amino acid pairs, each identified amino acid pair is calculated an F-score value, which indicates the significance of predicting glycosylation sites. To encode the amino acid pairs, a binary dimension in the features vector is set to 1 if a protein sequence contains the significant amino acid pair. Based on the results of the *k*-fold cross-validation, the ranked significant amino acid pairs can be added into the features vector one by one (forward feature selection) until the predictive performance is maximized.

### Model learning and evaluation

In this work, we adopted the QuickRBF package [18] to construct radial basis function network (RBFN) classifiers. As presented in Additional file 1, Figure S1, a general RBFN consists of three layers, namely the input layer, the hidden layer, and the output layer. The input layer broadcasts the coordinates of the input vector to each of the nodes in the hidden layer. Each node in the hidden layer then produces an activation based on the associated radial basis kernel function. Finally, each node in the output layer computes a linear combination of the activations of the hidden nodes. The general mathematical form of the output nodes in RBFN is as follows:

(2) $$c_{j}(x)=\sum_{i=1}^{k}w_{ji}\varphi (\left\|x-\mu_{i}\right\|;\sigma_{i});$$

where *c_j_*(*x*) denotes the function corresponding to the *j*-*th *output node and is a linear combination of *k *radial basis functions *ϕ*() with center μ_*i *_and bandwidth σ_*i*_; Also, w_*ji *_denotes the weight associated with the correlation between the *j*-*th *output node and the *i*-*th *hidden node. In this work, we adopted a fixed bandwidth (σ) of 5, and used all input nodes as centers (*k *= n). With its several bioinformatics applications, classification based on radial basis function (RBF) network has been extensively adopted to predict factors such as the cleavage sites in proteins [19], inter-residue contacts [20], protein disorder [21], and discrimination of β-barrel proteins [22].

Predictive performance of the constructed models is evaluated by performing five-fold cross validation. The five-fold cross validation has been performed with the sequence level. The 239 glycoproteins are divided into five approximately equal sized subgroups, about 48 sequences in each subgroup. In one round of cross-validation, a subgroup is regarded as the test set, and the remaining four subgroups are regarded as the training set. The RBFN classifier has been done for each fold separately using the data on training set and evaluated with the test set. The cross-validation process is repeated five rounds, with each of five subgroups used as the test set in turn. Then, the five results are combined to produce a single estimation. The advantage of five-fold cross-validation is that all original data are regarded as both training set and test set, and each data is used for test exactly once [23]. The following measures of predictive performance of the trained models are defined. Precision (Pr) = TP/(TP+FP), Sensitivity (Sn) = TP/(TP+FN), Specificity (Sp) = TN/(TN+FP), Accuracy (Acc) = (TP + TN)/(TP+FP+TN+FN), Balanced Accuracy (BAcc) = (Sn+Sp)/2, and

$$\text{Matthews Correlation Coefficient }\left(\text{MCC}\right)=\frac{\left(TP\times TN)-(FN\times FP\right)}{\sqrt{\left(TP+FN)\times (TN+FP)\times (TP+FP)\times (TN+FN\right)}},$$

where TP, TN, FP and FN denote the number of true positives, true negatives, false positives and false negatives, respectively. Additionally, predictive models are constructed for an independent test by using the window size and features that yield the highest accuracy.

## Results and discussions

### Distribution of O-linked glycosylation sites on transmembrane proteins

Based on the annotation of a membrane topology in UniProt [13], the distribution of glycosylated sites on transmembrane (TM) proteins is investigated. Table 2 summarizes the statistics of structural topology on 40 TM proteins. Six structural topology types are Lumenal (L), Nucleoplasmic (N), Extracellular (E); Cytoplasmic (C), Transmembrane (TM), and Signal peptide (S). For instance, as a nuclear membrane protein, Lamin-B receptor contains eight transmembrane segments which cover 27.4% of the sequence length. Table 3 displays the distribution of O-linked glycosylation sites on TM proteins. It is observed that nearly all of the O-linked glycosylation sites are located in the extracellular region of cell membrane proteins (87.6%). Furthermore, several O-linked glycosylation sites are located in luminal and nucleoplasmic regions which are the structural topology of non cell membrane proteins. According to the statistics of our dataset, no glycosylation sites are located on transmembrane regions as these could not be enzymatically accessed by glycotransferases. Therefore, the transmembrane regions of a protein can be considered to reduce the false positive of glycosylation site prediction.

**Table 2:** The distribution of structural topology on 40 transmembrane proteins that are extracted from release 15.0 of UniProt.

UniProt ID	Sequence length	Protein name	Number of TM segment	Percentage of sequence length in specific structural topology						
				L	N	E	C	TM	S	Unknown
O08984	620	Lamin-B receptor	TM = 8	0.0%	34.4%	0.0%	0.0%	27.4%	0.0%	38.2%
O14786	923	Neuropilin-1	TM = 1	0.0%	0.0%	90.5%	4.8%	2.5%	2.3%	0.0%
O15431	190	High affinity copper uptake protein 1	TM = 3	0.0%	0.0%	33.7%	33.2%	33.2%	0.0%	0.0%
P01375	233	Tumor necrosis factor	TM = 1	0.0%	0.0%	76%	15%	9%	0.0%	0.0%
P01589	272	Interleukin-2 receptor alpha chain	TM = 1	0.0%	0.0%	80.5%	4.8%	7%	7.7%	0.0%
P01867	404	Ig gamma-2B chain C region	TM = 1	0.0%	0.0%	86.6%	8.9%	4.5%	0.0%	0.0%
P02724	150	Glycophorin-A	TM = 1	0.0%	0.0%	48%	24%	15.3%	12.7%	0.0%
P02725	133	Glycophorin-A	TM = 1	0.0%	0.0%	46.6%	39.0.0%	17.3%	0.0%	0.0%
P02726	120	Glycophorin-A	TM = 1	0.0%	0.0%	40.8%	40.0%	19.2%	0.0%	0.0%
P02727	129	Glycophorin-A	TM = 1	0.0%	0.0%	50.4%	20.2%	16.3%	13.2%	0.0%
P02786	760	Transferrin receptor protein 1	TM = 1	0.0%	0.0%	88.4%	8.8%	2.8%	0.0%	0.0%
P03138	389	Large envelope protein	TM = 4	0.0%	0.0%	63.2%	14.7%	22.1%	0.0%	0.0%
P04441	279	H-2 class II histocompatibility antigen gamma chain	TM = 1	0.0%	0.0%	80.3%	10.4%	9.3%	0.0%	0.0%
P04921	128	Glycophorin-C	TM = 1	0.0%	0.0%	44.5%	36.7%	18.8%	0.0%	0.0%
P05067	770	Amyloid beta A4 protein	TM = 1	0.0%	0.0%	88.6%	6.1%	3.1%	2.2%	0.0%
P06028	91	Glycophorin-B	TM = 1	0.0%	0.0%	44%	11%	24.2%	20.9%	0.0%
P07204	575	Thrombomodulin	TM = 1	0.0%	0.0%	86.4%	6.3%	4.2%	3.1%	0.0%
P07359	626	Platelet glycoprotein Ib alpha chain	TM = 1	0.0%	0.0%	78.1%	16%	3.4%	2.6%	0.0%
P07725	236	T-cell surface glycoprotein CD8 alpha chain	TM = 1	0.0%	0.0%	69.1%	11%	8.9%	11%	0.0%
P08514	1039	Integrin alpha-IIb	TM = 1	0.0%	0.0%	92.6%	1.9%	2.5%	3%	0.0%
P08592	770	Amyloid beta A4 protein	TM = 1	0.0%	0.0%	88.6%	6.1%	3.1%	2.2%	0.0%
P11279	417	Lysosome-associated membrane glycoprotein 1	TM = 1	84.9%	0.0%	0.0%	2.9%	5.5%	6.7%	0.0%
P13473	410	Lysosome-associated membrane glycoprotein 2	TM = 1	84.6%	0.0%	0.0%	2.7%	5.9%	6.8%	0.0%
P13838	378	Leukosialin	TM = 1	0.0%	0.0%	59.6%	32.8%	6.1%	1.9%	0.0%
P14221	144	Glycophorin-A	TM = 1	0.0%	0.0%	47.9%	36.1%	16.0.0%	0.0%	0.0%
P16150	400	Leukosialin	TM = 1	0.0%	0.0%	58.2%	31%	5.8%	4.8%	0.0%
P17404	335	Chondromodulin-1	TM = 1	0.0%	0.0%	0.0%	0.0%	6.3%	0.0%	93.7%
P21583	273	Kit ligand	TM = 1	0.0%	0.0%	69.2%	13.2%	8.4%	9.2%	0.0%
P42098	421	Zona pellucida sperm-binding protein 3	TM = 1	0.0%	0.0%	85.3%	4.5%	5.0.0%	5.2%	0.0%
P51681	352	C-C chemokine receptor type 5	TM = 7	0.0%	0.0%	26.1%	27.0.0%	46.9%	0.0%	0.0%
P61073	352	C-X-C chemokine receptor type 4	TM = 7	0.0%	0.0%	281%	29.8%	42.0.0%	0.0%	0.0%
P80370	383	Protein delta homolog 1	TM = 1	0.0%	0.0%	73.1%	14.6%	6.3%	6.0.0%	0.0%
Q00657	2326	Chondroitin sulfate proteoglycan 4	TM = 1	0.0%	0.0%	94.4%	3.3%	1.1%	1.2%	0.0%
Q01455	230	Membrane protein	TM = 3	17.0.0%	0.0%	0.0%	57.4%	25.7%	0.0%	0.0%
Q07287	536	Zona pellucida sperm-binding protein 4	TM = 1	0.0%	0.0%	91.4%	0.7%	3.9%	3.9%	0.0%
Q09163	385	Protein delta homolog 1	TM = 1	0.0%	0.0%	73.2%	14.5%	6.2%	6.0.0%	0.0%
Q16790	459	Carbonic anhydrase 9	TM = 1	0.0%	0.0%	82.1%	5.2%	4.6%	8.1%	0.0%
Q62765	843	Neuroligin-1	TM = 1	0.0%	0.0%	77.3%	14.8%	2.5%	5.3%	0.0%
Q71M36	566	Chondroitin sulfate proteoglycan 5	TM = 1	0.0%	0.0%	69.4%	21.6%	3.7%	5.3%	0.0%
Q99075	208	Proheparin-binding EGF-like growth factor	TM = 1	0.0%	0.0%	67.8%	11.5%	11.5%	9.1%	0.0%

Abbreviation: L, Lumenal; N, Nucleoplasmic; E, Extracellular; C, Cytoplasmic; TM, Transmembrane; S, Signal peptide.

**Table 3:** The structural distribution of O-linked glycosylation sites on 40 transmembrane proteins that are extracted from release 15.0 of UniProt.

Type of membrane topology	Number of O-linked glycosylation sites
Extracellular	177

Lumenal	22

Nucleoplasmic	1

Cytoplasmic	0

Transmembrane	0

Unknown	2

### Characterization of O-linked glycosylation sites on transmembrane proteins and non-transmembrane proteins

This work focuses on analyzing O-linked glycosylated serine and threonine residues on TM proteins and non-TM proteins. Following the removal of homologous sequence of glycosylation sites, as shown in Table 4, flanking amino acids (-14 ~ +14) of the non-homologous glycosylated serine and threonine residues (glycosylation site centered on position 0) are graphically visualized as sequence logos. WebLogo [24,25] is adopted to generate the graphical sequence logo for the relative frequency of the corresponding amino acid at each position around the glycosylated sites. The conservation of amino acids that surround the glycosylation sites can then be easily examined. Based on the sequence logo representation, no amino acids around the modified sites are obviously conserved. However, several motifs are slightly varied between glycosylated TM and non-TM proteins. Thus, the flanking regions of glycosylation sites are examined to understand that the significant amino acid pairs differ between TM proteins and non-TM proteins, based on the F-score measurement.

**Table 4:** The sequence frequency logos of O-linked glycosylated serine and threonine on transmembrane (TM) proteins and non-transmembrane (non-TM) proteins that are extracted from release 15.0 of UniProt.

Glycosylated residues	Number of non-homologous sites	Number of proteins	Window length	Sequence frequency logo
Glycosylated serine on TM proteins	87	29	-14 ~ +14	

Glycosylated serine on non-TM proteins	281	27	-14 ~ +14	

Glycosylated threonine on TM proteins	115	118	-14 ~ +14	

Glycosylated threonine on non-TM proteins	356	137	-14 ~ +14	

Table 5 displays the identified significant amino acid pairs (SAAPs) flanking the O-linked glycosylated serine and threonine residues on the transmembrane proteins and non-transmembrane proteins. Each SAAP has a corresponding F-score value, implying that a higher value of F-score has more significant conservation in a specific dataset. For instance, the pair (+3T, +9E or +9T) suggests that the threonine (T) on position +3 and the glutamic acid (E) or threonine (T) on position +9 are significant with F-score 0.071 that surround O-linked glycosylated serine on transmembrane proteins. For the glycosylated TM proteins, 16 SAAPs and 25 SAAPs surround the O-linked glycosylated serine and threonine residues, respectively. For non-TM proteins, 32 SAAPS and 13 SAAPS are identified for O-linked glycosylated serine and threonine residues, respectively. All of the identified SAAPs are combined with the sequence of amino acids (BLOSUM62) to increase the prediction accuracy of O-linked glycosylation sites.

**Table 5:** The significant amino acid pairs that surround the O-linked glycosylated serine and threonine on transmembrane (TM) proteins and non-transmembrane (non-TM) proteins that are extracted from release 15.0 of UniProt.

O-linked glycosylated serine				O-linked glycosylated threonine			
TM proteins		non-TM proteins		TM proteins		non-TM proteins	
**Pair**	**F-score**	**Pair**	**F-score**	**Pair**	**F-score**	**Pair**	**F-score**

(+3T, +9E or +9T)	0.071	(+1G,-2E)	0.058	(+5 S,-9 M or -9P)	0.057	(+3P,-1P')	0.078
(-7T,+2 S or +3S)	0.064	(+1G,-1G)	0.041	(+5 S,-7T or -7S)	0.053	(+1P,-1P')	0.058
(+5P,+3P or +3T)	0.064	(+1G,+11G)	0.041	(+5P,+9E or +9P)	0.05	(+3P,+1P')	0.053
(+1T,+4P)	0.056	(+1G,+5G)	0.04	(-7T,-6 S or -6T)	0.049	(-1P,+5P')	0.053
(+1P,-1P)	0.056	(+1G,-3G)	0.034	(+5E,+1P or +1T or +1V)	0.047	(-4T,+3P')	0.036
(-7P,-5T)	0.053	(+1G,+10S)	0.034	(+7T,+8 S or +8P)	0.047	(+1P,+5P')	0.033
(+1G,+8E)	0.049	(+4 S,+5G)	0.032	(-7T,+8T)	0.044	(-4T,-1P')	0.033
(-7T,-12A or -12S)	0.047	(-1G,-2E)	0.031	(+7T,+10P or +10S)	0.043	(-4T,+14T')	0.033
(-7T,+6T)	0.047	(-5 S,+12S)	0.029	(-7T,-12S)	0.042	(-4T,+4T')	0.032
(+1G,-13D)	0.046	(-1P,-3P)	0.028	(+5P,-9T or -9V)	0.042	(+3T,+2A')	0.031
(+5P,-12S)	0.046	(+4 S,+1G)	0.027	(+1 S,-8 S or -8T)	0.041	(-4T,-10T')	0.030
(-7P,-10 S or -10T)	0.045	(+1G,+2E)	0.027	(+1T,+11 S or +11P)	0.041	(-4T,+2T')	0.030
(+3P,+11S)	0.043	(-5 S,+9A)	0.027	(+7P,+12 S or +12T)	0.040	(-1A,+3P')	0.030
(-7G,+6K or +6H)	0.038	(+9A,-5S)	0.027	(-7 S,+10S)	0.039		
(+3T,+10I)	0.036	(+1T,-7T)	0.026	(+7 S,+10S)	0.039		
(+1G,-1G)	0.034	(+4P,-3P)	0.025	(+1P,-13Q or -13T)	0.037		
		(-1G,-3G)	0.025	(-7P,-11 S or -11L)	0.036		
		(-1P,+3P)	0.025	(+1P,-3P or -3S)	0.036		
		(-5G,-4D)	0.025	(+5T,+10 S or +10E)	0.036		
		(-5 S,+2S)	0.024	(+5E,-7 S or -7T)	0.036		
		(+9A,+12S)	0.024	(+7P,+6D)	0.035		
		(-5 S,-12T)	0.023	(+1 S,-6 S or -6T)	0.034		
		(-1G,+9G)	0.021	(-7 S,-13P)	0.033		
		(-5 S,-7S)	0.019	(+1T,-13L or -13D)	0.030		
		(-1G,+7S)	0.018	(-10P,+7P or +7E)	0.017		
		(+4 S,+7S)	0.017				
		(+4 S,+12S)	0.017				
		(+4 S,-2E)	0.017				
		(-1G,+4S)	0.017				
		(-5G,-6S)	0.017				
		(+4 S,+14S)	0.016				
		(-5G,+10S)	0.015				

### Predictive performance of cross-validation

In this work, 29-mer (-14 ~ +14) is selected as the window length in the following evaluation and implementation. Initially, the sequence of amino acids around O-linked glycosylation sites is encoded using BlOSUM62 matrix and is evaluated in terms of predictive performance. Without the classification of TM proteins and non-TM proteins, the estimated sensitivity, specificity, accuracy, and balanced accuracy are 58.8%, 84.5%, 83.3%, and 71.6%, respectively. According to Table 6, the predictive sensitivity, specificity, accuracy, and balanced accuracy of glycosylated TM proteins are 65.3%, 81.1%, 79.9%, and 73.2% respectively. Notably, the negative set of training data is markedly larger than the positive one, thus, necessitating a consideration of the balanced accuracy for the skewed dataset. For O-linked glycosylated non-TM proteins, the predictive sensitivity, specificity, accuracy, and balanced accuracy are 56.7%, 85.1%, 83.9%, and 70.9%, respectively.

**Table 6:** The five-fold cross-validation performance of O-linked glycosylation sites on transmembrane (TM) proteins and non-transmembrane (non-TM) proteins that are extracted from release 15.0 of UniProt.

Training features	Amino acid (BLOSUM62)		Amino acid + SAAPs		Amino acid + SAAPs + Membrane topology*
	
	TM proteins	non-TM proteins	TM proteins	non-TM proteins	TM proteins
True Positive	132	361	132	384	132

False Positive	**462**	2124	365	1933	**317**

True Negative	1988	12110	2085	12301	2133

False Negative	70	276	70	253	70

Sensitivity	65.3%	56.7%	65.3%	60.3%	65.3%

Specificity	81.1%	85.1%	85.1%	86.4%	87.1%

Accuracy	79.9%	83.9%	83.6%	85.3%	85.4%

Balanced Accuracy	73.2%	70.9%	75.2%	73.4%	76.2%

MCC	0.30	0.23	0.34	0.26	0.37

*This process considers the structural topology of transmembrane in the prediction of O-linked glycosylation sites on transmembrane proteins.

Combining the significant amino acid pairs with the sequence of amino acids increases the predictive specificity for glycosylated membrane proteins to 85.1%. The accuracy and balanced accuracy are improved without affecting the estimated sensitivity. For non-TM proteins, both predictive sensitivity and specificity increased to 60.3% and 86.4%, respectively. Consequently, according to the evaluation of the five-fold cross validation, the identified significant amino acid pairs can increase the predictive performance of GlycoRBF.

### Effects of considering membrane topology on transmembrane proteins

This work also investigates the various motifs of O-linked glycosylation sites between TM proteins and non-TM proteins. For TM proteins, the membrane topology can be considered to further increase the prediction accuracy. As for statistics of O-linked glycosylation sites on TM proteins (Table 3), the transmembrane region is assumed to reduce the number of false positive predictions. As shown in Table 6, false positive predictions are reduced from 462 to 317, thereby reducing false positive data by 31.4% as compared to models that do not consider membrane topology. Since O-linked glycosylation sites must be identified from a novel protein sequence, an effective membrane topology prediction tool, MEMSAT-SVM [26], was firstly applied to discriminate between TM and non-TM proteins and annotate the structural topology on TM proteins.

### Predictive performance of an independent test

Models are evaluated whether they are over-fitted to their training data or not. This is done by constructing and using independent sets of data concerning O-linked glycosylated sites on TM proteins and non-TM proteins to demonstrate the effectiveness of the RBF models, which have the highest prediction accuracy. As presented in Table 7, the balanced accuracies of the proposed method are 60.1% and 70.9% when it is applied to glycosylated TM proteins and non-TM proteins, respectively. With 14 O-linked glycosylation sites and 1238 non-glycosylation sites on TM proteins, the proposed model can achieve a sensitivity of 50.0% and a specificity of 70.2%. For non-TM proteins, there are 44 O-linked glycosylation sites and 662 non-glycosylation sites, and the proposed model can achieve a sensitivity of 61.4% and a specificity of 80.4%. This analysis reveals that the performance in an independent test is comparable to that of the cross-validation.

**Table 7:** Comparison of independent test between GlycoRBF and other methods.

Methods		GlycoRBF	GPP	NetOglyc3.1	CKSAAP
O-linked glycosylation sites on TM proteins	Sensitivity	50.0%	57.1%	21.4%	28.6%
	
	Specificity	70.2%	38.4%	82.1%	74.2%
	
	Accuracy	70.0%	38.7%	81.4%	73.6%
	
	Balanced Accuracy	**60.1%**	47.8%	51.7%	51.4%
	
	MCC	0.05	-0.01	0.01	0.01

O-linked glycosylation sites on non-TM proteins	Sensitivity	61.4%	54.5%	47.7%	20.5%
	
	Specificity	80.4%	44.1%	82.0%	78.2%
	
	Accuracy	79.2%	44.8%	79.9%	74.6%
	
	Balanced Accuracy	**70.9%**	49.3%	64.9%	49.4%
	
	MCC	0.24	-0.01	0.18	-0.01

Other O-linked glycosylation predictors were tested based on independent test sets. According to the results, GPP [11] has a high estimated sensitivity in identifying O-linked glycosylation sites but has a low estimated specificity when applied to independent test sets of TM proteins. For non-TM proteins, GPP has a balanced performance between sensitivity and specificity. NetOglyc3.1 [2] and CKSAAP [10] have poor sensitivity in identifying O-linked glycosylation sites, but have high specificity. Owing to a large size of non-glycosylation site, NetOglyc3.1, which has high estimated specificity, outperforms other methods in terms of prediction accuracy. While considering balanced accuracy, this method which specifies the degree of significance outperforms other approaches in term of predicting O-linked glycosylation sites. Also, according to [27], we have performed paired t-test as our statistical significance test during different approaches. The p-value of paired t-test between our proposed method and other methods are 0.024, 0.036, and 0.035, respectively. The p-values show that our proposed method is statistically different than other methods.

### Implementation of the prediction scheme

**Figure 2:**
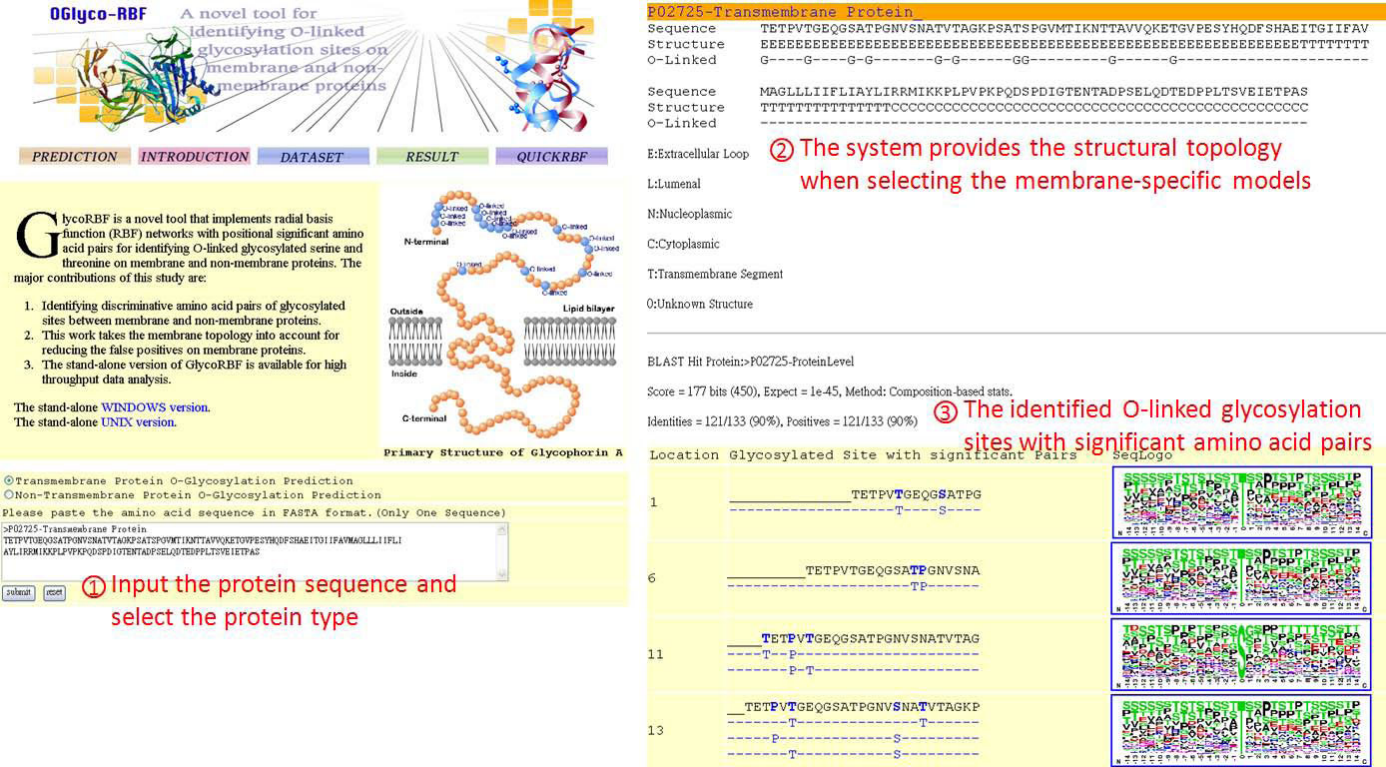
**The user interface of web-based GlycoRBF**.

In utilizing time-consuming and laboratory-intensive experimental identification of protein glycosylation sites, although glycosylated proteins can be identified, precisely identifying the glycosylated sites on the substrate is difficult. Therefore, an effective prediction scheme should be developed to efficiently identify potential O-linked glycosylation sites. Following evaluation by cross-validation and an independent test, amino acid sequences (BLOSUM62) and the significant amino acid pairs are utilized to construct RBF models in order to predict the O-linked glycosylation of serine and threonine. As shown in Figure 2, users can submit their uncharacterized protein sequences and select a specific TM protein whose structural topology is considered. The system, named GlycoRBF, returns the predictions efficiently, including O-linked glycosylated position, the flanking amino acids, significant amino acid pairs, and the sequence logo. In particular, the system provides the structural topology when users select TM-specific models for predicting the O-linked glycosylation sites. The stand-alone version of GlycoRBF is also available for high throughput data analysis.

**Figure 3:**
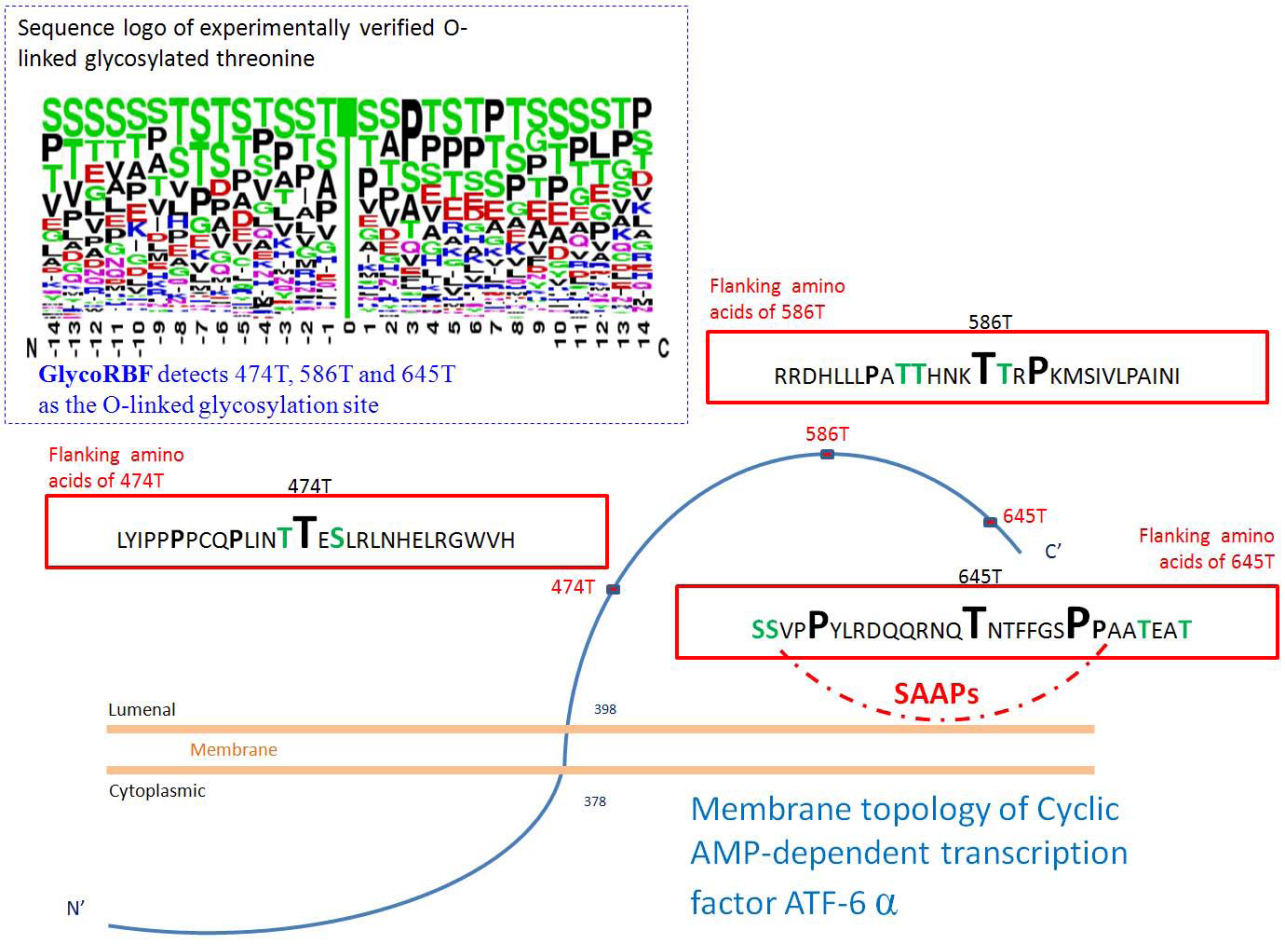
**A case study of human cyclic AMP-dependent transcription factor ATF-6 alpha that contains three O-linked glycosylation sites at 474T, 586T, and 645T, based on the annotation of HPRD**.

To demonstrate the performance of GlycoRBF, a case study was presented. Cyclic AMP-dependent transcription factor ATF-6 alpha, which is located in the endoplasmic reticulum membrane, has been proposed that under ER stress the cleaved N-terminal cytoplasmic domain translocates into the nucleus [28]. As shown in Figure 3, cyclic AMP-dependent transcription factor ATF-6 alpha was experimentally confirmed that contains three O-linked glycosylation sites at 474T, 586T, and 645T [29]. With the annotation of structural topology, GlycoRBF could specifically reduce the false positives and accurately identify the true glycosylated threonine at position 474, 586, and 645. The conserved amino acids round 474T and 586T are colored in Figure 3, based on the sequence logo of experimentally verified O-linked glycosylation site of threonine. Moreover, the significant amino acid pair, that was used to identify O-linked glycosylated 645T, is also provided.

### Evaluation of physicochemical properties around the glycosylation sites

**Figure 4:**
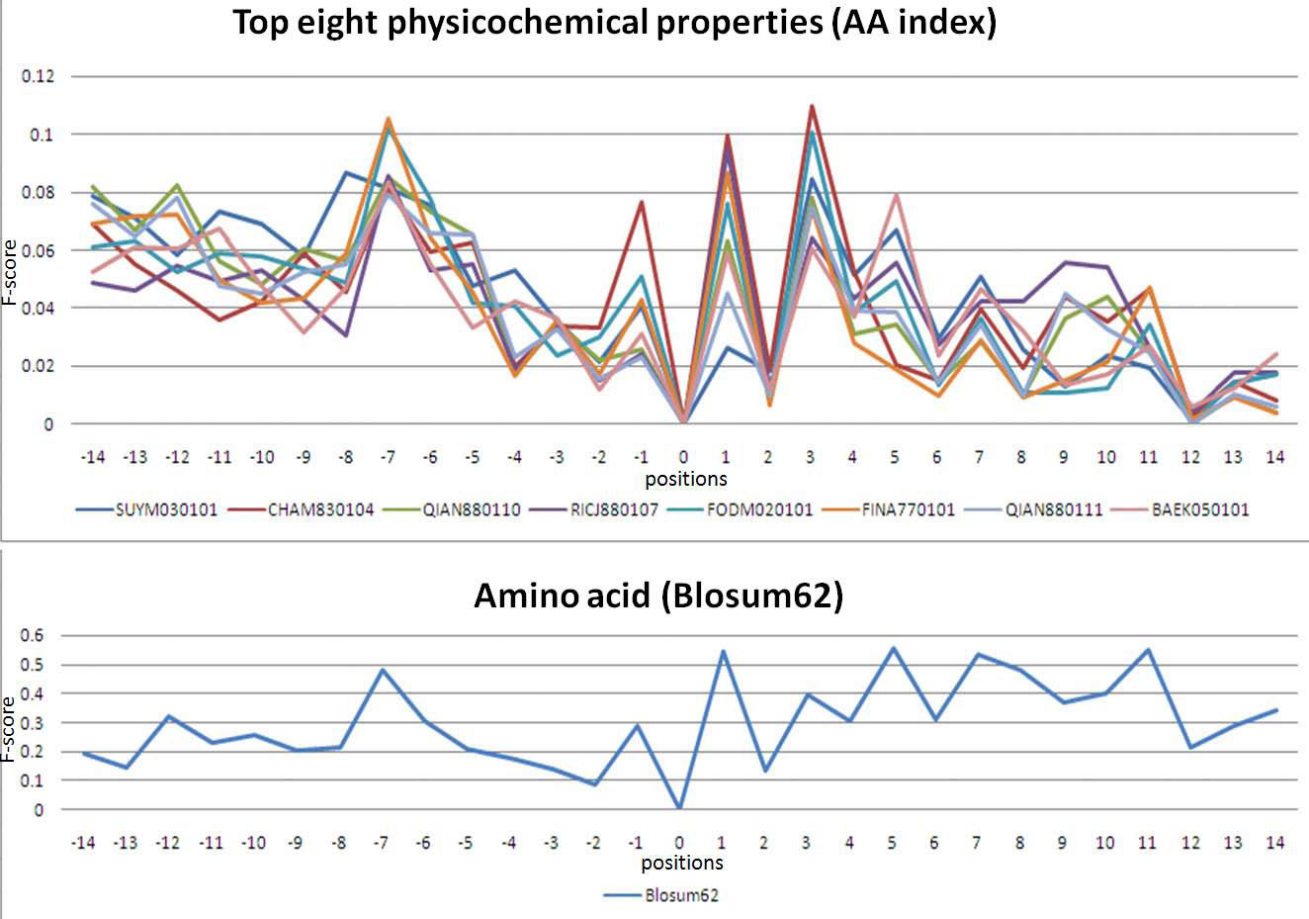
**The *F*-score measurement of top eight physicochemical properties surrounding the O-linked glycosylation sites of transmembrane proteins**.

Most predictive models are based on the features of amino acid sequences. However, previous work has utilized 31 informative physicochemical properties to identify protein ubiquitylation sites [30]. To investigate the characteristics of O-linked glycosylation sites in a comprehensive manner, 531 physicochemical properties extracted from version 9.1 of AAindex [12] are evaluated to distinguish the glycosylation sites from the non-glycosylation sites. Based on the measurement of *F*-score, Figure 4 indicates that eight physicochemical properties around O-linked glycosylation sites have significantly differential values at position -7, -1, +1, and +3 (glycosylation site centered on position 0), which have a similar significance to BLOSUM62-coded amino acids. For glycosylated TM proteins, the significant physicochemical properties include linker propensity index [31], weights for alpha-helix at the window position of 3 [32], relative preference value at N4 [33], propensity of amino acids within pi-helices [34], helix-coil equilibrium constant [35], weights for alpha-helix at the window position of 4 [32], and linker index [36]. Each significant physicochemical property is combined with the feature of amino acid sequences for evaluation of the predictive performance using five-fold cross-validation. As shown in Table S1 (Additional file 1), the predictive performance of integrating physicochemical properties is similar to the model that only uses the feature of amino acid sequences.

In the case of glycosylated non-TM proteins, the significant physicochemical properties consist of the number of bonds in the longest chain [37], absolute entropy, volume [38], side chain volume [39], radius of gyration of side chain [40], average volume of buried residue [41], and residue volume [42,43]. Table S2 (Additional file 1) shows that the combination of physicochemical property and amino acid sequence performs slightly worse than the model that is only trained with the feature of amino acid sequences. To further investigate the functions of O-linked glycosylated non-TM proteins, the functional annotations that are obtained from Gene Ontology [44] are listed in Table S3 (Additional file 1). Most of the glycosylated non-TM proteins are associated with blood coagulation, inflammatory response, immune response, cell adhesion, and transporter.

## Conclusions

O-linked glycosylation prediction methods in previous studies, including NetOglyc3.1 [2], CKSAAP [10], and GPP [11], do not consider glycosylated protein types. However, the proposed scheme incorporates significant amino acid pairs (SAAPs) to increase the prediction accuracy of O-linked glycosylated sites on transmembrane and non-transmembrane proteins. Based on the method of RBF networks, the cross-validation accuracies of O-linked glycosylated sites on TM proteins and non-TM proteins are 83.6% and 85.1%, respectively. When the structural topology on glycosylated TM proteins is considered, the prediction accuracy can reach 85.4%, subsequently reducing false positives by 31.4%. Comparison of the performance of the proposed approach with that of previous methods reveals that GlycoRBF has the significantly higher prediction accuracy according to independent testing. Additionally, GlycoRBF is implemented as an effective web server for predicting the O-linked glycosylation sites on TM proteins and non-TM proteins. A case study of Cyclic AMP-dependent transcription factor ATF-6 alpha was presented to demonstrate the effectiveness of GlycoRBF. The stand-alone version of GlycoRBF is also available for high throughput data analysis.

Although the proposed method can perform accurately and robustly, based on the results of independent tests, some issues must still be addressed in future work. First, there are numerous sugars found in glycoproteins including xylose, fucose, galactose, glucose, mannose, N-acetylglucosamine, N-acetylgalactosamine, and N-acetylneuraminic acid [1]. Various catalytic enzymes involve glycotransferases, sugar-transferring enzymes and glycosidases, which trim specific monosaccharides from precursors to form intermediate structures [3]. Therefore, future research should further investigate the characteristics of O-linked glycosylation sites according to the sugar types attached on glycosylated sites. Second, future research should examine the structural preferences of glycosylated sites in greater detail, especially in O-linked glycosylated serine and threonine, whose flanking residues are not conserved. In addition to the solvent accessible surface area, secondary structure, B-factor, intrinsic disordered region, protein linker region, and other factors at experimental O-linked glycosylation sites that are located in the protein regions with PDB entries, should be studied. Finally, the independent test sets that are proposed herein are blind to the trained model during cross-validation, but may not be to previously proposed predictors. Hence, developing a benchmark for constructing test sets that are truly independent of each predictor is a worthwhile task.

## Availability

GlycoRBF can be accessed via a web interface, and is freely available to all interested users at http://GlycoRBF.bioinfo.tw. The stand-alone version of GlycoRBF is also available for high throughput data analysis. All of the data set that is used in this work is also available.

## Authors' contributions

TYL and YYO conceived and supervised the project. SAC was responsible for the design, computational analyses, implemented the web-based tool, and draft the manuscript with revisions provided by TYL and YYO. All authors read and approved the final manuscript.

## Supplementary Material

Additional file 1 **Figure S1**. The general architecture of RBFN consisting of input layer, hidden layer, and output layer. **Table S1**. The predictive performance of significant physicochemical properties in glycosylated transmembrane proteins. **Table S2**. The predictive performance of significant physicochemical properties in glycosylated non-transmembrane proteins. **Table S3**. Functional analysis of glycosylated non-transmembrane proteins. **Table S4**. Independent dataset of transmembrane protein. **Table S5**. The distribution of O-linked glycosylation sites on transmembrane proteins of independent test set. Click here for file
