# Supplementary material for: Incorporating significant amino acid pairs to identify O-linked glycosylation sites on transmembrane proteins and non-transmembrane proteins
Source: BMC Bioinformatics. 2010 Oct 29;11:536. doi: 10.1186/1471-2105-11-536 (PMC2989983; doi:10.1186/1471-2105-11-536)
Supplement: Additional file 1 — Figure S1. The general architecture of RBFN consisting of input layer, hidden layer, and output layer. Table S1. The predictive performance of significant physicochemical properties in glycosylated transmembrane proteins. Table S2. The predictive performance of significant physicochemical properties in glycosylated non-transmembrane proteins. Table S3. Functional analysis of glycosylated non-transmembrane proteins. Table S4. Independent dataset of transmembrane protein. Table S5. The distribution of O-linked glycosylation sites on transmembrane proteins of independent test set. [file 1471-2105-11-536-S1.DOC]

**Supplementary Materials**

**Figure S1.** The general architecture of RBFN consisting of input layer, hidden layer, and output layer.


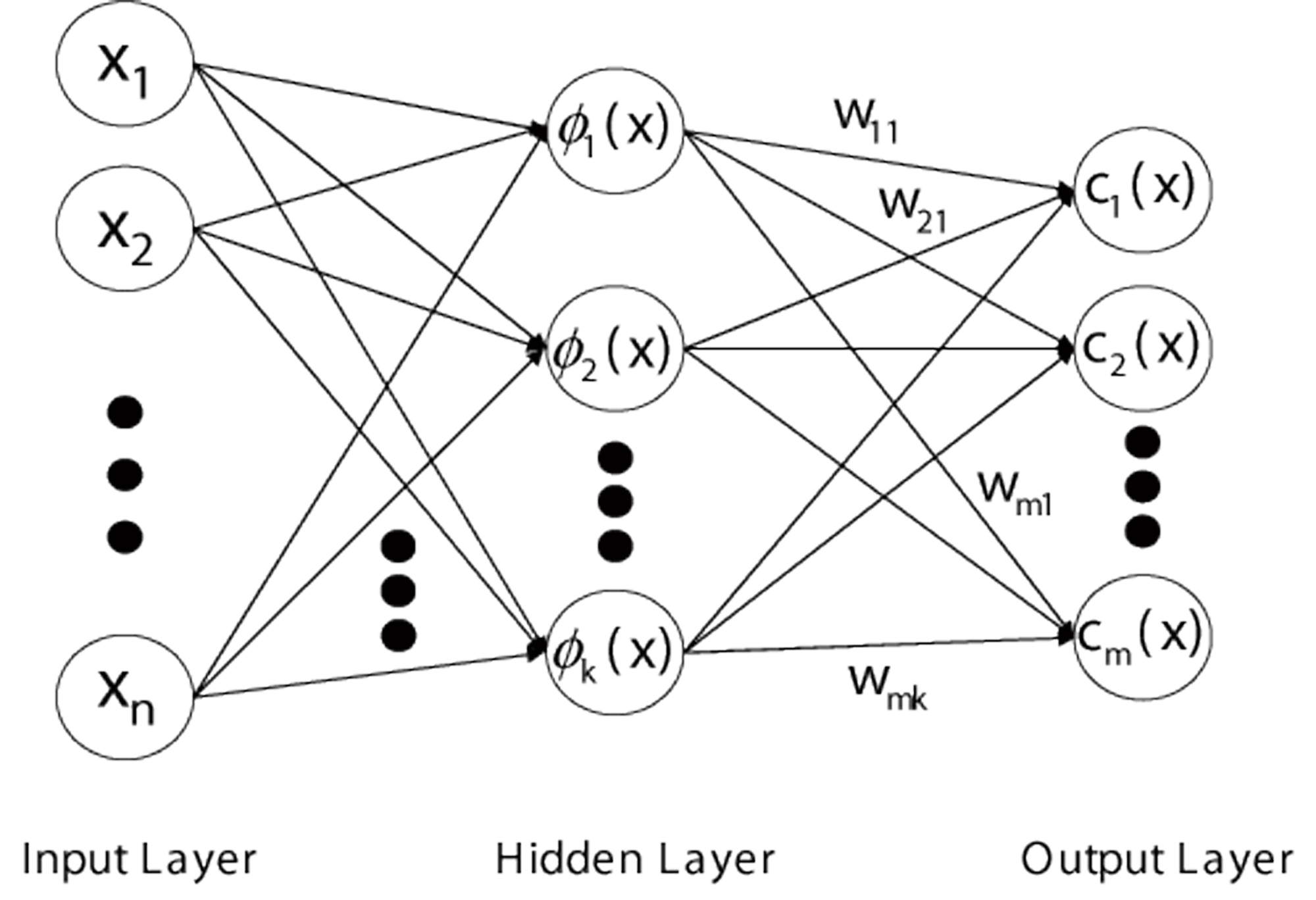


**Table S1**. The predictive performance of significant physicochemical properties in glycosylated transmembrane proteins.

| **Features** | **AAindex ID** | **Reference** | **Membrane proteins** | | | |
| --- | --- | --- | --- | --- | --- | --- |
|  |  |  | **Sn** | **Sp** | **Acc** | **Bacc** |
| Blosum62 | - | - | 59.9% | 83.7% | 81.9% | 71.8% |
| Blosum62+ Linker propensity index | **SUYM030101** | Suyama-Ohara, 2003 | 61.9% | 83.5% | 81.9% | 72.7% |
| Blosum62+Weights for alpha-helix at the window position of 3 | QIAN880110 | Qian-Sejnowski, 1988 | 58.9% | 83.6% | 81.7% | 71.3% |
| Blosum62+ Relative preference value at N4 | **RICJ880107** | Richardson-Richardson, 1988 | 60.4% | 83.3% | 81.6% | 71.9% |
| Blosum62+ Propensity of amino acids within pi-helices | FODM020101 | Fodje-Al-Karadaghi, 2002 | 59.4% | 83.7% | 81.8% | 71.6% |
| Blosum62+ Helix-coil equilibrium constant | FINA770101 | Finkelstein-Ptitsyn, 1977 | 60.4% | 83.6% | 81.8% | 72.0% |
| Blosum62+ Weights for alpha-helix at the window position of 4 | QIAN880111 | Qian-Sejnowski, 1988 | 59.9% | 83.6% | 81.8% | 71.8% |
| Blosum62+ Linker index | BAEK050101 | Bae et al., 2005 | 57.9% | 84.0% | 82.0% | 71.0% |
| Blosum62+ Helix-coil equilibrium constant | PTIO830101 | Ptitsyn-Finkelstein, 1983 | 60.9% | 83.5% | 81.9% | 72.2% |

Abbreviation: Sn, sensitivity; Sp, specificity; Acc, accuracy; Bacc, balanced accuracy.

**Table S2**. The predictive performance of significant physicochemical properties in glycosylated non-transmembrane proteins.

| **Features** | **AAindex ID** | **Reference** | **Non-membrane proteins** | | | |
| --- | --- | --- | --- | --- | --- | --- |
|  |  |  | **Sn** | **Sp** | **Acc** | **Bacc** |
| Blosum62 | - | - | 59.8% | 85.5% | 84.4% | 72.7% |
| Blosum62+The number of bonds in the longest chain | CHAM830106 | Charton-Charton, 1983 | 57.0% | 85.9% | 84.7% | 71.5% |
| Blosum62+ Absolute entropy | HUTJ700102 | Hutchens, 1970 | 57.3% | 86.0% | 84.8% | 71.7% |
| Blosum62+ Volume | GRAR740103 | Grantham, 1974 | 57.0% | 85.9% | 84.7% | 71.5% |
| Blosum62+ Side chain volume | KRIW790103 | Krigbaum-Komoriya, 1979 | 56.7% | 85.9% | 84.6% | 71.3% |
| Blosum62+ Radius of gyration of side chain | LEVM760105 | Levitt, 1976 | 57.0% | 85.9% | 84.7% | 71.5% |
| Blosum62+ Average volume of buried residue | CHOC750101 | Chothia, 1975 | 56.8% | 85.8% | 84.6% | 71.3% |
| Blosum62+ Residue volume | BIGC670101 | Bigelow, 1967 | 56.5% | 85.8% | 84.6% | 71.2% |
| Blosum62+ Residue volume | GOLD730102 | Goldsack-Chalifoux, 1973 | 56.7% | 85.8% | 84.6% | 71.3% |

Abbreviation: Sn, sensitivity; Sp, specificity; Acc, accuracy; Bacc, balanced accuracy.

**Table S3. Functional analysis of glycosylated non-transmembrane proteins.**

| **UniProt ID** | **Recommended Name** | **Biological Process** | **Signaling Pathway** |
| --- | --- | --- | --- |
| A2AQ25 | Sickle tail protein; |  |  |
| O08537 | Estrogen receptor beta; | transcription; | estrogen receptor signaling pathway; |
| O16883 | Chondroitin proteoglycan 4; |  |  |
| O74213 | Polygalacturonase 1; | cell wall biogenesis/degradation; |  |
| O88737 | Protein bassoon; |  |  |
| O88778 | Protein bassoon; |  |  |
| O88935 | Synapsin-1; |  |  |
| O95972 | Bone morphogenetic protein 15; |  |  |
| P00740 | Coagulation factor IX; | blood coagulation; |  |
| P00741 | Coagulation factor IX; | blood coagulation; |  |
| P00742 | Coagulation factor X; | blood coagulation; |  |
| P00743 | Coagulation factor X; | blood coagulation; |  |
| P00744 | Vitamin K-dependent protein Z; | blood coagulation; |  |
| P00747 | Plasminogen; | blood coagulation;tissue remodeling; |  |
| P00748 | Coagulation factor XII; | blood coagulation; |  |
| P00749 | Urokinase-type plasminogen activator; | blood coagulation;chemotaxis;plasminogen activation; |  |
| P00750 | Tissue-type plasminogen activator; | blood coagulation;plasminogen activation; |  |
| P00999 | Seminal plasma acrosin inhibitor A1; |  |  |
| P01042 | Kininogen-1; | blood coagulation;inflammatory response; |  |
| P01044 | Kininogen-1; | blood coagulation;inflammatory response; |  |
| P01045 | Kininogen-2; | blood coagulation;inflammatory response; |  |
| P01106 | Myc proto-oncogene protein; | transcription; |  |
| P01172 | Somatostatin-2; |  |  |
| P01189 | Pro-opiomelanocortin; |  | neuropeptide signaling pathway; |
| P01190 | Pro-opiomelanocortin; |  | neuropeptide signaling pathway; |
| P01217 | Glycoprotein hormones alpha chain; |  |  |
| P01233 | Choriogonadotropin subunit beta; | apoptosis; |  |
| P01344 | Insulin-like growth factor II; | carbohydrate metabolism;osteogenesis; | insulin receptor signaling pathway; |
| P01374 | Lymphotoxin-alpha; |  |  |
| P01563 | Interferon alpha-2; | antiviral defense;inflammatory response; | cell surface receptor linked signaling pathway; |
| P01588 | Erythropoietin; | erythrocyte maturation; |  |
| P01876 | Ig alpha-1 chain C region; | immune response; |  |
| P01878 | Ig alpha chain C region; |  |  |
| P01880 | Ig delta chain C region; | immune response; |  |
| P02470 | Alpha-crystallin A chain; |  |  |
| P02488 | Alpha-crystallin A chain; |  |  |
| P02505 | Alpha-crystallin A chain; |  |  |
| P02649 | Apolipoprotein E; | lipid transport; |  |
| P02656 | Apolipoprotein C-III; | lipid transport;lipid degradation; |  |
| P02668 | Kappa-casein; |  |  |
| P02732 | Ice-structuring glycoprotein 3; |  |  |
| P02749 | Beta-2-glycoprotein 1; | plasminogen activation; |  |
| P02750 | Leucine-rich alpha-2-glycoprotein; |  |  |
| P02751 | Fibronectin; | acute phase;angiogenesis;cell adhesion;cell shape; |  |
| P02760 | Protein AMBP; | cell adhesion;host-virus interaction; |  |
| P02765 | Alpha-2-HS-glycoprotein; | mineral balance; |  |
| P02777 | Platelet factor 4; | chemotaxis;immune response; | cytokine-mediated signaling pathway; |
| P02784 | Seminal plasma protein PDC-109; | fertilization; |  |
| P02787 | Serotransferrin; | iron transport; |  |
| P02790 | Hemopexin; | host-virus interaction; |  |
| P02810 | Salivary acidic proline-rich phosphoprotein 1/2; |  |  |
| P02974 | Fimbrial protein; | cell adhesion; |  |
| P03395 | Envelope glycoprotein; |  |  |
| P04141 | Granulocyte-macrophage colony-stimulating factor; | immune response; |  |
| P04180 | Phosphatidylcholine-sterol acyltransferase; | lipid metabolism; |  |
| P04278 | Sex hormone-binding globulin; |  |  |
| P04963 | Chloroperoxidase; |  |  |
| P05059 | Chromogranin-A; |  |  |
| P05155 | Plasma protease C1 inhibitor; | blood coagulation;immune response; |  |
| P05431 | Fimbrial protein; | cell adhesion; |  |
| P05451 | Lithostathine-1-alpha; |  |  |
| P05452 | Tetranectin; |  |  |
| P05783 | Keratin, type I cytoskeletal 18; | cell cycle;host-virus interaction; |  |
| P06027 | Echinoidin; |  |  |
| P06765 | Platelet factor 4; | chemotaxis;immune response; | cytokine-mediated signaling pathway; |
| P06867 | Plasminogen; | blood coagulation;tissue remodeling; |  |
| P06868 | Plasminogen; | blood coagulation;tissue remodeling; |  |
| P06870 | Kallikrein-1; |  |  |
| P07498 | Kappa-casein; |  |  |
| P07585 | Decorin; |  |  |
| P07589 | Fibronectin; | acute phase;angiogenesis;cell adhesion;cell shape; |  |
| P07898 | Aggrecan core protein; |  |  |
| P07987 | Exoglucanase 2; | carbohydrate metabolism; |  |
| P07996 | Thrombospondin-1; | apoptosis;cell adhesion;immune response; |  |
| P08318 | Large structural phosphoprotein; |  |  |
| P08709 | Coagulation factor VII; | blood coagulation; |  |
| P08751 | Lutropin/choriogonadotropin subunit beta; |  |  |
| P09951 | Synapsin-1; |  |  |
| P0C828 | Kappa-A-conotoxin SIVA; |  |  |
| P10124 | Serglycin; | apoptosis;biomineralization; |  |
| P10451 | Osteopontin; | biomineralization;cell adhesion; |  |
| P10493 | Nidogen-1; | cell adhesion; |  |
| P10645 | Chromogranin-A; |  |  |
| P10646 | Tissue factor pathway inhibitor; | blood coagulation; |  |
| P11831 | Serum response factor; | transcription; |  |
| P12021 | Apomucin; |  |  |
| P12027 | Polysialoglycoprotein; |  |  |
| P12108 | Collagen alpha-2(IX) chain; |  |  |
| P12729 | Prespore-specific protein A; |  |  |
| P12763 | Alpha-2-HS-glycoprotein; | mineral balance; |  |
| P12839 | Neurofilament medium polypeptide; |  |  |
| P13501 | C-C motif chemokine 5; | cell adhesion;chemotaxis;exocytosis;immune response;inflammatory response; | |
| P13727 | Bone marrow proteoglycan; | immune response; |  |
| P14210 | Hepatocyte growth factor; |  |  |
| P15522 | Glycosylation-dependent cell adhesion molecule 1; |  |  |
| P17955 | Nuclear pore glycoprotein p62; | mrna transport;protein transport; | cell surface receptor linked signaling pathway; |
| P18684 | Diptericin-D; | immune response; |  |
| P18774 | Fimbrial protein; |  |  |
| P19527 | Neurofilament light polypeptide; |  |  |
| P19785 | Estrogen receptor; | transcription; |  |
| P19823 | Inter-alpha-trypsin inhibitor heavy chain H2; |  |  |
| P19827 | Inter-alpha-trypsin inhibitor heavy chain H1; |  |  |
| P19835 | Bile salt-activated lipase; | lipid degradation; |  |
| P20840 | Alpha-agglutinin; | cell adhesion; |  |
| P21793 | Decorin; |  |  |
| P21799 | Endocuticle structural glycoprotein ABD-4; |  |  |
| P21809 | Biglycan; |  |  |
| P21810 | Biglycan; |  |  |
| P22457 | Coagulation factor VII; | blood coagulation; |  |
| P22891 | Vitamin K-dependent protein Z; | blood coagulation; |  |
| P23928 | Alpha-crystallin B chain; |  |  |
| P24593 | Insulin-like growth factor-binding protein 5; |  |  |
| P24807 | Signal transducer CD24; |  | cell surface receptor linked signaling pathway; |
| P25236 | Selenoprotein P; |  |  |
| P26213 | Polygalacturonase-1; | cell wall biogenesis/degradation; |  |
| P26631 | Hirullin-P18; |  |  |
| P27918 | Properdin; | immune response; |  |
| P28314 | Peroxidase; | hydrogen peroxide; |  |
| P28512 | Hirudin-P6; |  |  |
| P30034 | Platelet factor 4; | chemotaxis;immune response; | cytokine-mediated signaling pathway; |
| P31096 | Osteopontin; | biomineralization;cell adhesion; |  |
| P32781 | A-agglutinin-binding subunit; | cell adhesion; |  |
| P36193 | Drosocin; | immune response; |  |
| P36912 | Endo-beta-N-acetylglucosaminidase F2; |  |  |
| P36913 | Endo-beta-N-acetylglucosaminidase F3; |  |  |
| P37199 | Nuclear pore complex protein Nup155; | mrna transport;protein transport; |  |
| P37362 | Pyrrhocoricin; | immune response; |  |
| P39060 | Collagen alpha-1(XVIII) chain; | cell adhesion; |  |
| P39873 | Brain ribonuclease; |  |  |
| P40225 | Thrombopoietin; |  |  |
| P41996 | Chondroitin proteoglycan-2; | cell cycle; |  |
| P47001 | Cell wall mannoprotein CIS3; | cell wall biogenesis/degradation; |  |
| P48304 | Lithostathine-1-beta; |  |  |
| P51671 | Eotaxin; | cell adhesion;chemotaxis;immune response;inflammatory response; | |
| P54684 | Lebocin-1/2; | immune response; |  |
| P54939 | Talin-1; |  |  |
| P55067 | Neurocan core protein; |  |  |
| P55796 | Lebocin-3; | immune response; |  |
| P57039 | Fimbrial protein; | cell adhesion; |  |
| P57672 | Vespulakinin-1; |  |  |
| P60568 | Interleukin-2; | cell adhesion;immune response; |  |
| P69327 | Glucoamylase; | carbohydrate metabolism; |  |
| P69328 | Glucoamylase; | carbohydrate metabolism; |  |
| P79119 | Epiphycan; |  |  |
| P80060 | Protease inhibitors; |  |  |
| P80195 | Glycosylation-dependent cell adhesion molecule 1; |  |  |
| P81019 | Seminal plasma protein BSP-30 kDa; | fertilization; |  |
| P81054 | Peptidyl-Lys metalloendopeptidase; |  |  |
| P81121 | Seminal plasma protein HSP-1; | fertilization; |  |
| P81428 | Trocarin; | blood coagulation; |  |
| P81437 | Formaecin-2; | immune response; |  |
| P81438 | Formaecin-1; | immune response; |  |
| P81447 | Glycosylation-dependent cell adhesion molecule 1; |  |  |
| P81577 | Cuticle protein AM1199; |  |  |
| P81578 | Cuticle protein AM1239; |  |  |
| P81579 | Cuticle protein AM1274; |  |  |
| P81755 | Epsilon conotoxin TxVA; |  |  |
| P81824 | Platelet-aggregating proteinase PA-BJ; |  |  |
| P83427 | Heliocin; | immune response; |  |
| P83762 | Submaxillary mucin; |  |  |
| P84293 | Hemocyanin subunit 2; | oxygen transport; |  |
| P84883 | GPI-anchored glycoprotein NETNES; |  |  |
| P84902 | Cassiicolin; |  |  |
| P85800 | Variegin; |  |  |
| Q00001 | Rhamnogalacturonase A; | cell wall biogenesis/degradation; |  |
| Q01172 | Pectin lyase A; |  |  |
| Q05819 | Heparin lyase I; |  |  |
| Q12127 | Covalently-linked cell wall protein 12; | cell wall biogenesis/degradation; |  |
| Q14624 | Inter-alpha-trypsin inhibitor heavy chain H4; | acute phase; |  |
| Q16627 | C-C motif chemokine 14; | immune response; |  |
| Q17802 | Chondroitin proteoglycan 1; | cell cycle; |  |
| Q21175 | Chondroitin proteoglycan 8; |  |  |
| Q29011 | Aggrecan core protein; | cell adhesion; |  |
| Q46079 | Chondroitinase-B; |  |  |
| Q47899 | Flavastacin; |  |  |
| Q4KLH5 | Arf-GAP domain and FG repeats-containing protein 1; | differentiation; |  |
| Q50906 | Alanine and proline-rich secreted protein apa; |  |  |
| Q59288 | Chondroitinase-AC; |  |  |
| Q62261 | Spectrin beta chain, brain 1; |  |  |
| Q7M4E9 | Endocuticle structural glycoprotein SgAbd-3; |  |  |
| Q7M4F0 | Endocuticle structural glycoprotein SgAbd-9; |  |  |
| Q7M4F1 | Endocuticle structural glycoprotein SgAbd-4; |  |  |
| Q7M4F2 | Endocuticle structural glycoprotein SgAbd-8; |  |  |
| Q7M4F3 | Endocuticle structural glycoprotein SgAbd-2; |  |  |
| Q7M4F4 | Endocuticle structural glycoprotein SgAbd-1; |  |  |
| Q7TQD2 | Tubulin polymerization-promoting protein; |  |  |
| Q7YWX9 | Chondroitin proteoglycan 7; |  |  |
| Q80Z38 | SH3 and multiple ankyrin repeat domains protein 2; |  |  |
| Q86NG3 | C-type lectin domain-containing protein 88; |  |  |
| Q8BMB0 | Protein EMSY; | dna damage;transcription; |  |
| Q8IZD2 | Histone-lysine N-methyltransferase MLL5; | cell cycle;transcription; | retinoic acid receptor signaling pathway; |
| Q95114 | Lactadherin; | angiogenesis;cell adhesion;fertilization; |  |
| Q95NH6 | Attacin-C; | immune response; |  |
| Q95XP7 | Chondroitin proteoglycan 9; |  |  |
| Q9C1S9 | Exoglucanase-6A; | carbohydrate metabolism; |  |
| Q9MZ06 | Fibroblast growth factor-binding protein 1; |  |  |
| Q9QYX7 | Protein piccolo; |  |  |
| Q9XVS3 | C-type lectin domain-containing protein 87; |  |  |
| Q9XYR5 | Contulakin-G; |  |  |

**Table S4.** Independent dataset of transmembrane protein.

| **Uniprot ID** | **Length** | **Recommended Name** | **Protein Structure percentage**  **(L,N,E,C,T,S,Non)** | | | | | | | **Count of TM Segments** |
| --- | --- | --- | --- | --- | --- | --- | --- | --- | --- | --- |
| O88393 | 850 | TGF-beta receptor type III | 0.0% | 0.0% | 89.8% | 4.9% | 2.7% | 2.6% | 0.0% | TM=1 |
| P18828 | 311 | Syndecan-1 | 0.0% | 0.0% | 74.9% | 11.3% | 6.8% | 7.1% | 0.0% | TM=1 |
| Q14242 | 412 | P-selectin glycoprotein ligand 1 | 0.0% | 0.0% | 73.5% | 17.2% | 5.1% | 4.1% | 0.0% | TM=1 |
| Q7M750 | 143 | Opalin | 0.0% | 0.0% | 21.0% | 64.3% | 14.7% | 0.0% | 0.0% | TM=1 |
| 00388_1 | 554 | Macrophage colony-stimulating factor 1 | 83.8% | 0.0% | 0.0% | 6.7% | 3.8% | 5.8% | 0.0% | TM=1 |
| 01628_1 | 440 | Secretin receptor(GPCR 2 Family) | 0.0% | 0.0% | 38.2% | 21.6% | 35.2% | 5.0% | 0.0% | TM=7 |
| 02667_1 | 700 | Meprin A subunit beta | 0.0% | 0.0% | 90.0% | 3.9% | 3.0% | 3.1% | 0.0% | TM=1 |
| 11829_1 | 2169 | Mucin-4 | 0.0% | 0.0% | 0.0% | 0.0% | 1.0% | 1.3% | 97.7% | TM=1 |
| 16118_1 | 670 | Cyclic AMP-dependent transcription factor ATF-6 alpha | 40.6% | 0.0% | 0.0% | 56.3% | 3.1% | 0.0% | 0.0% | TM=1 |

**Table S5.** The distribution of O-linked glycosylation sites on transmembrane proteins of independent test set.

| **Membrane topology** | **Number of O-liked glycosylation sites** |
| --- | --- |
| Extracellular | 9 |
| Lumenal | 4 |
| Nucleoplasmic | 0 |
| Cytoplasmic | 0 |
| Transmembrane | 0 |
| Unknown | 1 |
